# Comment on Gjestland, T. A Systematic Review of the Basis for WHO’s New Recommendation for Limiting Aircraft Noise Annoyance. *Int. J. Env. Res. Pub. Health* 2018, *15*, 2717

**DOI:** 10.3390/ijerph16071088

**Published:** 2019-03-27

**Authors:** Rainer Guski, Dirk Schreckenberg, Rudolf Schuemer, Mark Brink, Stephen A. Stansfeld

**Affiliations:** 1 Ruhr-University Bochum, Psychology Department, 44801 Bochum, Germany; 2 ZEUS GmbH, Zentrum für Angewandte Psychologie, Umwelt- und Sozialforschung, Sennbrink 46, 58093 Hagen, Germany; schreckenberg@zeusgmbh.de; 3 Independent Researcher, 58095 Hagen, Germany; ar-schuemer@t-online.de; 4 Federal Office for the Environment, 3003 Bern, Switzerland; Mark.Brink@bafu.admin.ch; 5 Centre for Psychiatry, Wolfson Institute of Preventive Medicine, Barts and the London School of Medicine, Queen Mary University of London, London EC1M 6BQ, UK; s.a.stansfeld@qmul.ac.uk

## Abstract

In his recent discussion paper in this journal, Truls Gjestland attempts a “systematic review”, as he calls it, of the evidence base for aircraft noise annoyance, consolidated in a meta-analysis by Guski et al. that informed the recommended guideline value of 45 dB *L*_den_ in the recently published World Health Organization (WHO) Environmental Noise Guidelines. He questions the validity of the presented evidence, as “some of the referenced studies have not been conducted according to standardized methods, and the selection of respondents is not representative of the general airport population.” Gjestland maintains that the new WHO Guidelines are based on a questionable selection of existing aircraft noise studies. Our reply comments on the arguments of Gjestland and refutes most of his critique.

## 1. On the Selection of Original Studies

On page 2, Gjestland [1] comments on the inclusion/exclusion of studies in the systematic review by Guski et al. [2] (carrried out for the WHO Environmental Noise Guidelines for the European Region [3]) as follows: “*For the three excluded studies the authors could not find a regression function that they could use to estimate % HA [Highly Annoyed]*.” For carrying out the meta-analysis, we asked all authors of identified original studies to provide equations or parameter estimates of their exposure–response relationships. Indeed, the authors of three studies did not provide the requested information, and these studies were thus excluded from the calculation of a common exposure–response function.

In the next paragraph, Gjestland goes on with “One of these studies [Gjestland omits giving a reference] comprises surveys at two different airports, but Guski et al. only considered one of them for reasons unknown.” If the study of Gelderblom et al. [4] is meant here, the two surveys in that study in fact took place at two different airports, Trondheim and Bodo. The latter is a combined civil and military airport, which we judged not to be comparable with the rest of the airports selected for the meta-analysis (which so far contained only civil airports). Gjestland continues: “It is interesting to note that results from previous similar surveys at both of these airports which were excluded, were included in the analysis by Miedema & Vos for their well-known EU reference curve.” Yes, this is an astonishing fact, given that the perception of military aircraft noise is probably difficult to describe in terms of *L*_eq_, because military airports are characterized by relatively few overflights and usually very high noise levels per event. For us, this was reason enough to exclude studies involving military aircraft noise (see Section 2.2 in our systematic review).

## 2. On Non-Acoustic Factors

Later in his paper, Gjestland writes: “A procedure based on combining all responses from different surveys in this manner represents [a] simple way of analyzing data from aircraft noise annoyance surveys. It ignores the fact that only about one third of the variance in the response data is explained by the cumulative noise exposure … and it effectively prohibits any possibility of studying the influence of non-acoustic factors ….” We reject both allegations as nonsensical. First, of course, a whole range of variables, besides *L*_den_, are contributing to individual annoyance judgments. This is mentioned in chapter 3.1.7. In the same chapter, it is explained that our review does not handle individual (within-study) variability, it does handle between study characteristics, such as study quality rating, survey type, noise level range, response rate, and rate of airport change. Second, why does our way of analyzing secondary data “effectively prohibit any possibility of studying the influence of non-acoustic factors”? This is nonsense, proposing a common exposure–response curve does not prohibit anything.

On page 3, Gjestland writes “The WHO Guideline Development Group also seems to be ignorant about the importance of non-acoustic factors. In the Guidelines publication it is stated that ‘in noise annoyance studies non-acoustic factors may explain up to 33% of the variance’. This must be a misunderstanding. The correct statement should be that acoustic factors (or rather *L*_den_-based factors) may explain up to 33% of the variance, while the other two-thirds are explained by non-acoustic factors.” Gjestland is wrong here: acoustic factors (or rather *L*_den_-based factors) may explain up to 33% of the variance, while the other two-thirds are variance induced by non-acoustic factors (another 33%) and (another 33%) remaining non-explained (error) variance.

Later on, on page 4, Gjestland suspects that the aircraft accident at Milano Linate two years before the HYENA (HYpertension and Exposure to Noise near Airports) survey took place, might have left marks on residents around Malpensa airport and increased their annoyance: “*High fear of accidents has been found to shift the annoyance response equivalent to as much as 20 dB in the exposure.*” Today, the scientific evidence for this effect is rather scarce or just anecdotal. Gjestland quotes work from the late 1990s, and most of the more recent studies do not include “fear of accidents” questions. It seems that this variable has lost its importance for affecting annoyance responses. However, even if it would be an important predictor, the accident at Linate airport could have affected annoyance responses at other airports (beyond Malpensa) as well and could have also affected responses many years later. Basically, the temporal or spatial range of the effect of an accident at one airport on responses at another airport is unknown.

## 3. On Age Effects on Annoyance

At the end of page 3 in his paper, Gjestland sets about to criticize the inclusion of the HYENA study [5] in our meta-analysis. He starts with the observation that the HYENA study included respondents aged 45–70 years only. He then mentions the van Gerven et al. [6] review, which reported a nonlinear relation in the form of an inverted “U” between age and annoyance, and draws the conclusion that the HYENA sample may be biased towards higher annoyance compared to samples aged 20–80. Indeed, the van Gerven paper shows quite drastic effects of age on annoyance. However, other more recent studies do not or report only small effects. For instance, the NORAH (Noise Related Annoyance, Cognition and Health) study reports a very weak nonlinear effect of age (eta-square between 0.01 and 0.03, see Schreckenberg et al. [7], figure A-20). Brink et al. [8] found no significant effects of age and age squared on aircraft noise annoyance in a fully adjusted exposure–response model. This said, we do not see why the exclusion of respondents younger than 45 years in the HYENA sub-studies should qualify these sub-studies to be excluded from our meta-analysis.

Gjestland then writes: “Guski et al. are aware that this fact has most certainly contributed to an increase in annoyance in the HYENA study, but still they choose to include the data in violation of their own selection criteria (‘member of the general population’)”. If Gjestland means “representative of the general population”, this was not an inclusion criterion, and of course persons aged 45 and more are still members of the general population.

## 4. On Low-Rate Change and High-Rate-Change Airports (Studies)

On page 4, Gjestland writes: “someone that has endured a noisy construction period of perhaps 3 to 4 years and then suddenly has been exposed to unfamiliar aircraft noise for two years, cannot be considered a typical airport neighbor.” We agree that the construction and implementation of a new airport is a rare and noisy event, and one of the major components defining a “high rate change airport”. However, is there any civil airport which does not carry out construction work regularly? Also, is there any common definition of a “representative European airport”? Is there any widely shared definition of a “typical airport neighbor”? We believe the answer is “No” to all of these questions.

Gjestland mentions the exclusion of the Athens and Milan airports from the pooled exposure–annoyance curve in the Babisch et al. [5] paper: *“They [Babisch et al.] discuss several reasons for this and conclude that the data from these two airports are not representative for airports in general.”* Should we thus have excluded the Athens and Milan sub-studies from our meta-analysis? Babisch et al. [5] do not conclude that the data from these two airports are not representative for airports in general. Rather, they wrote (p. 1175): “*The airport-specific annoyance curves gave some indication that the very high annoyance scoring of the Athens and Milan sample could have been due to an overshooting of annoyance reporting (over-reaction) because of recent changes in airport operations” … “Furthermore, based on the assessment of noise sensitivity, there was some indication that a certain degree of selection bias (more noise sensitive subjects in more aircraft noise exposed areas) might have been present in the Milan sample. We therefore excluded these two airports from the pooled analyses.*” We included both airports because we wanted to include all studies that met our inclusion criteria. This led to a share of 63% study participants in high-rate change (HRC) conditions, which was discussed in Sections 3.1.4 and 3.1.8 in (see [2] main text), and in (see [2] supplementary materials, sections S4, S10, and S13). We did indeed not exclude any study based on a speculation about the presence of selection bias.

## 5. On the Effect of Weighting Studies for Pooling Evidence

**Figure 1:**
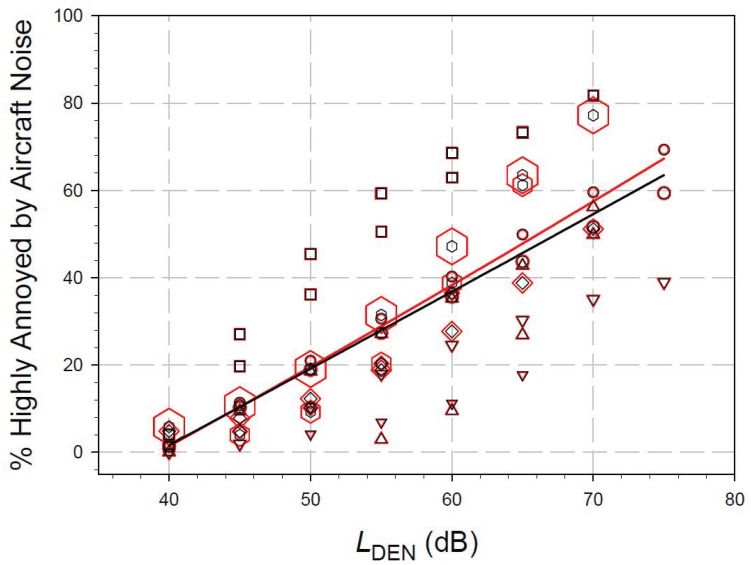
Exposure–response curves for aircraft noise annoyance responses in the WHO dataset [2]. “Highly annoyed” refers to respondents using ≥73% of the annoyance response scale. The red data points and regression line refer to study size weighting according to sample size; the black data points and curve refer to the same dataset without study size weighting.

Gjestland criticized the weighting of studies according to study size in our meta-analysis. At first sight, he seems to be right that weighting can induce bias instead of reducing it and that, for example, the quite large Amsterdam–Schiphol study (*N* = 5873, cp. [9]) had a large and therefore disproportionate influence on the final common exposure–response function. However, if one wants to estimate a common effect size with the highest precision and validity possible, one would weight studies according to study quality. In view of our goal to estimate a reliable mean effect size from a set of studies differing in effect size and sample size, we decided to weight studies according to sample size, which is a very common procedure in systematic reviews. It should be noted that we weighted the studies according to the square root of sample size. This procedure is a non-linear form of weighting which reduces the impact of the absolute sample size at larger sample sizes. The overall effect of this weighting approach is thus less “dramatic” than initially feared. In fact, the influence of sample size weighting on the WHO aircraft noise dataset is relatively small, as can be seen in Figure 1.

In Figure 1 it can be observed that below 50 dB *L*_den_ there is no relevant difference between the two curves. That is, the WHO Guideline Development Group’s conclusions regarding their recommendations in the WHO Environmental Noise Guidelines would not have been different if study size weighting were omitted in the meta-analysis.

## 6. On the Use of the Community Tolerance Level (CTL) Approach

**Figure 2:**
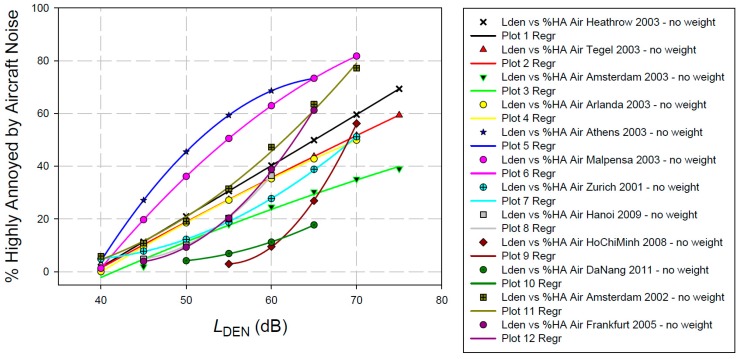
Individual exposure–response curves for aircraft noise annoyance responses in the 12 studies of the full WHO dataset [2]. % Highly Annoyed (%HA) refers to respondents using ≥73% of the annoyance response scale (same definition as in Figure 1). No weighting according to sample size is applied here.

On page 6, Gjestland proposes the use of exposure–response curves based on the CTL approach. One of the assumptions of the CTL approach is that the sigmoidal form and the slope of the exposure–annoyance relationship are fixed (cp. [10]). In other words, the CTL approach assumes that the form and slope of the exposure–response function is identical for all airports. This is contrary to our experience. For instance, if we put the individual exposure–response curves of each of the studies in the full WHO aircraft noise annoyance dataset into one plot, we observe a multitude of different shapes (see Figure 2).

In view of such differences between exposure–response curves from different annoyance studies (usually, as here, confounded with airports), it is very doubtful if a common curve can be derived at all. Taking into account the multitude of different exposure–response curves (and their shapes), we do not see any indication that the application of the CTL method would allow for a more reliable common exposure–response curve, even if the selection of studies would have been performed according to a more rigid protocol than has been done in Gjestland’s paper. Moreover, the derivation of a generalized exposure–response curve based on the CTL method cannot be recommended.

## 7. Conclusions

In this reply, we substantiated our decisions regarding the inclusion or exclusion of studies in our systematic review on aircraft noise annoyance [2]. We demonstrated that the common practice of weighting studies according to sample size has no unwanted side effects, and that the central assumption of the CTL approach contradicts empirical evidence: the form of the exposure–response relation is not fixed, it may differ from one study to another. All in all, we conclude that there were no specific flaws, faults, or inaccuracies in the analysis of the available evidence in the bespoke systematic review. We are convinced that the WHO Guideline Development Group did not come to false conclusions and that their recommended guideline value for aircraft noise is not unjustifiably low.

## References

[1] Gjestland T. (2018). A Systematic Review of the Basis for WHO’s New Recommendation for Limiting Aircraft Noise Annoyance. Int. J. Environ. Res. Pub. Health.

[2] Guski R., Schreckenberg D., Schuemer R. (2017). Review: WHO Environmental Noise Guidelines for the European Region: A Systematic Review on Environmental Noise and Annoyance. Int. J. Environ. Res. Pub. Health.

[3] World-Health-Organization-Europe (2018). Environmental Noise Guidelines for the European Region.

[4] Gelderblom F.B., Gjestland T.T., Granoien I.L.N., Taraldsen G. (2014). The Impact of Civil Versus Military Aircraft Noise on Noise Annoyance. Proceedings of the INTER-NOISE and NOISE-CON Congress and Conference.

[5] Babisch W., Houthuijs D., Pershagen G., Cadum E., Katsouyanni K., Velonakis M., Dudley M.L., Marohn H.D., Swart W., Breugelmans O. (2009). Annoyance due to aircraft noise has increased over the years—Results of the HYENA study. Environ. Int..

[6] Van Gerven P.W.M., Vos H., Van Boxtel M., Janssen S., Miedema H. (2009). Annoyance from environmental noise across the lifespan. J. Acoust. Soc. Am..

[7] Schreckenberg D., Faulbaum F., Guski R., Ninke L., Peschel C., Spilski J., Wothge J. (2015). Endbericht, Band 3: Wirkungen von Verkehrslärm auf die Belästigung und Lebensqualität. Verkehrslärmwirkungen im Flughafenumfeld.

[8] Brink M., Schäffer B., Vienneau D., Foraster M., Pieren R., Eze I.C., Cajochen C., Probst-Hensch N., Röösli M., Wunderli J.-M. (2019). A survey on exposure-response relationships for road, rail, and aircraft noise annoyance: Differences between continuous and intermittent noise. Environ. Int..

[9] Breugelmans O.R.P., van Wiechen C.M., van Kamp I., Heisterkamp S.H., Houthuijs D. (2004). Gezondheid en beleving van de omgevingskwaliteit in de regio Schiphol: 2002-Tussenrapportage Monitoring Gezondheidskundige Evaluatie Schiphol. Health and Quality of Life Near Amsterdam Schiphol Airport: 2002.

[10] Fidell S. (2018). A Modern Standardized Method for Predicting Community Response to Aircraft Noise. Civ. Eng. Archit..

